# Thermal and Electrical Characterization of Polyester Resins Suitable for Electric Motor Insulation

**DOI:** 10.3390/polym15061374

**Published:** 2023-03-09

**Authors:** Elisa Calabrese, Marialuigia Raimondo, Michelina Catauro, Luigi Vertuccio, Patrizia Lamberti, Raffaele Raimo, Vincenzo Tucci, Liberata Guadagno

**Affiliations:** 1 Department of Industrial Engineering, University of Salerno, Via Giovanni Paolo II, 132, 84084 Fisciano, Italy; 2 Department of Engineering, University of Campania “Luigi Vanvitelli”, Via Roma 29, 81031 Aversa, Italy; 3 Department of Information and Electrical Engineering and Applied Mathematics, University of Salerno, Via Giovanni Paolo II, 132, 84084 Fisciano, Italy

**Keywords:** insulating materials, polyester resins, thermal analyses, Vacuum Pressure Impregnation (VPI), PWM-fed motors

## Abstract

This paper undertakes the thermal and electrical characterization of three commercial unsaturated polyester imide resins (UPIR) to identify which among them could better perform the insulation function of electric motors (high-power induction motors fed by pulse-wide modulation (PWM) inverters). The process foreseen for the motor insulation using these resins is Vacuum Pressure Impregnation (VPI). The resin formulations were specially selected because they are one-component systems; hence, before the VPI process, they do not require mixing steps with external hardeners to activate the curing process. Furthermore, they are characterized by low viscosity and a thermal class higher than 180 °C and are Volatile Organic Compound (VOC)-free. Thermal investigations using Thermogravimetric Analysis (TGA) and Differential Scanning Calorimetry (DSC) techniques prove their excellent thermal resistance up to 320 °C. Moreover, impedance spectroscopy in the frequency range of 100 Hz–1 MHz was analyzed to compare the electromagnetic performance of the considered formulations. They manifest an electrical conductivity starting from 10^−10^ S/m, a relative permittivity around 3, and a loss tangent value lower than 0.02, which appears almost stable in the analyzed frequency range. These values confirm their usefulness as impregnating resins in secondary insulation material applications.

## 1. Introduction

Thermosetting resins are materials that, during their processing, undergo a molecular crosslinking process called the “curing stage”, which irreversibly modifies their structure, changing from viscous liquids to rigid and highly cross-linked polymer solids [1]. In the field of thermosetting materials, an important role is played by epoxy resins and unsaturated polyesters (UP), as they have a more compactly crosslinked structure that leads to better mechanical, thermal, and chemical resistance features than the other resins. Although all these advantageous aspects allow employing these thermosetting polymers as matrices for structural composites in several engineering applications (automotive, aircraft, and aerospace industries, etc.), their high crosslinking degree makes them intrinsically brittle, with poor resistance to crack initiation and propagation, limiting their development [2,3].

Concerning epoxy resins employed for structural composites, the main approach, proposed to overcome the non-trivial issue of poor impact damage resistance, has been the integration into the polymeric matrix of auto-repair functionality through the employment of different strategies, such as those based on the storage of a healing agent within microcapsules [4,5,6,7,8,9,10,11,12] and vascular networks [13,14] or those inspired by supramolecular chemistry [15,16,17,18,19].

Furthermore, both epoxy and polyester resins found relevant application in the impregnation industry due to their capacity to provide mechanical and electrical support to windings [20,21,22,23,24]. Their excellent electrical insulation properties have allowed the development of various epoxy-based [20,21,24] and polyester-based systems [20,22,23] for Vacuum Pressure Impregnation (VPI) insulation processing of generators, motors, transformers, and other electrical equipment.

Impregnation is a fundamental step of the manufacturing process of stators for electrical machines, and it fulfils different requirements, among which are the maximum resin penetration into the coil system, better heat dissipation, and endurance at higher operating temperatures, performant insulation, and the minimization of noise through the elimination of vibration. Impregnation improves the quality of insulation with a positive impact on the lifetime of the electrical machines [25,26].

Nevertheless, it is worth noting that in the presence of high amplitude, fast varying, time-dependent voltage supply, such as in high power induction motors fed by pulse-wide modulated (PWM) inverters, the insulation system is solicited by harmonics components of the electrical voltage supply. Such components may involve material dielectric losses, leading to degradation of the thermal performance and the reduction of its lifetime [27,28]. Therefore, the motor insulation system should present superior dielectric characteristics, especially at the switching frequency (fs) and at its multiple values, to withstand the elevated voltage gradients in the PWM environment. Owing to the fast developments in power electronics, this frequency easily reaches values such as 20 kHz. The higher the switching frequency, the faster the motor insulation degradation occurs. There is no simple correlation between the insulation life and the switching frequency. Nevertheless, many experiments show that the probability of insulation failure moves from a direct proportionality to a quadratic one when *fs* reaches a value equal to or higher than 5 kHz [29]. In particular, it is of fundamental importance from an application point of view to take into account the electrical material properties covering a wide frequency range (up to several multiples of the PWM fundamental frequency governed by the switching frequency) to consider a more suitable solution for improving the lifetime of the electrical machines. Together with the issues related to the electrical performance of the insulating coverings, other aspects to be taken into account are related to the chemical nature of the insulating polymer and the processing adopted for the insulation. The two main impregnation techniques are dipping and trickling. During the dipping procedure, the stator is wholly submerged and therefore surrounded by the resin, while in the trickling procedure, significant parts of the stator are specifically targeted by a jet of fluid resin. The most efficient method for the impregnation of insulating machines is considered the VPI dipping procedure, during which, in the first phase, the stator is dipped in the resin under vacuum conditions, followed by a second phase, in which the stator is soaked in resin and the pressure is further increased significantly above atmospheric conditions to eliminate the remaining air cavities inside the slots. Afterward, the stator with the impregnated resin is put in an oven at a specific temperature to cure the surrounding resin. This procedure improves the resin intrusion into the stator geometry, ensuring optimal insulation performance [25]. An important goal that the electrical manufacturing industry had to achieve in the development of resins intended for the impregnation process is the reduction of Volatile Organic Compound (VOC) content, causing severe issues of environmental pollution [30,31,32,33]. The method used to reach this target is the production of solventless-type resins, which include both unsaturated polyester and epoxy resins. Regarding polyester resins, the VOC amount is attributed to the toxic cross-linking monomer component of the resin, which has two main functions in the polymerization process: it acts as a solvent for the base polyester, reducing its viscosity, and it is reactive during the curing [23]. To meet air VOC emissions standards, the strategy adopted by some companies has been the reduction or the replacement of the toxic organic monomer, such as styrene or vinyl toluene, with nontoxic components, developing environmentally friendly polyester-imide impregnating resins with low viscosity and having excellent thermal performance [33]. A further fundamental aspect to consider in the development of resins for insulating systems is their thermal classification. For standard rotating machines, the thermal classification, determined by testing according to international standards (e.g., IEEE 1776 or IEC 60034-18-31 [34,35]), gives the maximum absolute temperature allowed during operating conditions. For example, thermal Class H means that temperatures of 180 °C can be reached without shortening the expected lifetime of the rotating machine [36]. Nowadays, one main objective is to develop resins for insulating systems with a thermal class equal to or higher than 180 °C.

Currently, there are three main families of resins used for the impregnation of insulating systems: polyesters, epoxies, and polyesterimide resins. The best candidates are polyesterimide-based resins, as polyester-based liquids are easy to use but have some mechanical criticalities (e.g., brittleness) and are dielectrically weak at high temperatures, and epoxy-based liquids achieve excellent mechanical, chemical, and thermal resistance, but usually have relatively high viscosity. Differently, polyesterimide-based liquids are characterized by a structural chemistry similar to polyesters (low viscosity). Still, they also have improved dielectric properties and thermal properties comparable to those of epoxy resins, exhibiting a thermal class of 180 °C [37]. Despite the relevant industrial interest in these resins, to the best of the authors’ knowledge, a comprehensive study considering thermal, electrical, and mechanical properties is missing in the literature. In light of this, the current work focuses on the thermal and electrical characterizations of three commercial unsaturated polyester imide resins, specially selected because they are one-component (no additive or hardener must be added), characterized by low viscosity and a thermal class higher than 180 °C, and Volatile Organic Compound (VOC)-free. These materials were first characterized by thermogravimetric analysis (TGA) and differential scanning calorimetry (DSC) techniques, which confirmed their efficiency in providing a preliminary evaluation of the thermal resistance of the studied materials [38,39]. Subsequently, electromagnetic characterization was performed, considering that the impregnating resins provide electrical insulation as a secondary insulation material in generators, electric motors, and transformers. Therefore, it is of fundamental importance, from an application point of view, to ensure an electrical characterization covering a wide frequency range. For this reason, we conducted a comparison of the considered commercial resins in terms of electromagnetic behavior by performing impedance spectroscopy between 100 Hz and 1 MHz. In particular, electrical conductivity, relative electrical permittivity, and loss tangent of the studied systems were analyzed. The comparison of the results allowed the identification of which investigated resins could perform better in electric motor insulation.

## 2. Materials and Methods

### 2.1. Materials

Three commercial resins developed for motor coils’ Vacuum Pressure Impregnation (VPI) were analyzed. These materials are one-component unsaturated polyester imide resins, indicated with the acronyms DAMISOL, VOTASTAT, and VOLTATEX (see Table 1).

These resins have an extremely low smell, and referring to the European directive 2010/75/EU, they are without any VOC, such as styrene, vinyl-toluene, or diallyl phthalate. Due to their high thermal resistance, they can be used on any electrical equipment exposed to high-temperature conditions. They are suitable for insulation systems up to thermal class 200. They show good resistance against solvent gases and good adhesion.

Before evaluating the thermal and electrical properties of the materials, the liquid resins were oven polymerized by employing the hardening process scheduled in the technical data sheets of the materials. The hardening conditions are summarized in Table 2.

**Figure 1:**
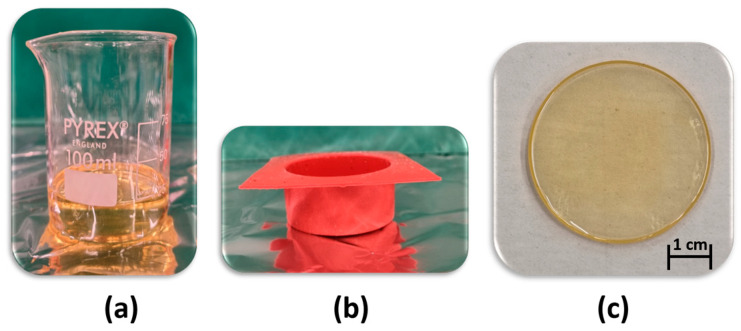
Pictures representing (**a**) liquid uncured DAMISOL resin; (**b**) circular silicon mold; (**c**) thermally cured DAMISOL resin with circular shape for electrical characterization.

To obtain the samples, about 2.2 g of liquid resin (see Figure 1a) was poured into a circular silicon mold with a diameter of 4 cm (see Figure 1b). Then, the material was oven cured, obtaining a rigid disk with a thickness of about 3.0 mm, shown in Figure 1c, and used to perform the electrical measurements.

The pictures of the obtained samples with the different commercial resins are reported in Figure S1 in the Supplementary Materials.

### 2.2. Thermal Characterization

Two types of thermal investigation were performed on the prepared samples, Differential Scanning Calorimetry (DSC) and Thermogravimetric Analyses (TGA). DSC analyses were carried out by using a thermal analyzer Mettler DSC 822/400 (Mettler-Toledo Columbus, OH, USA) equipped with a DSC cell purged with nitrogen and chilled with liquid nitrogen for sub-ambient measurements. DSC was employed to evaluate the samples’ curing degree (DC), assuming that exothermic heat developed during the curing process is proportional to the extent of the curing reactions. The DC can be determined from the total heat of reaction (Δ*H_T_*) of the curing reactions and the residual heat of reaction (Δ*H_Res_*) of the partially cured resin according to Equation (1) [40].

(1) $$DC=\frac{\Delta H_{T}-\Delta H_{Res}}{\Delta H_{T}}\times 100$$

The total heat of reaction (Δ*H_T_*) was determined by performing the DSC analysis on the liquid uncured resins, scanning about 7.0 mg of the sample by a heating run at 10 °C/min from 30 to 300 °C, while the Δ*H_Res_* was determined from the measurements performed on the oven-hardened samples, by scanning the polymerized materials at 10 °C/min from 30 °C to 300 °C.

TGA analyses were carried out using a Mettler TGA/SDTA 851 thermal analyzer and were performed in air flow. The weight loss as a function of the temperature was recorded at 10 °C/min from 30 to 900 °C.

### 2.3. Mechanical Characterization

Tensile tests were carried out using an INSTRON instrument (series 4301 INSTRON, Norwood, MA, USA) with a rate of 1 mm/min. The tests were executed at room temperature, with a relative humidity of 50%, and using samples with a rectangular geometry (3.0 × 100 × 25 mm^3^). Six specimens were tested for each sample, and the results are reported in Figure S2 in the Supplementary Materials.

### 2.4. Spectroscopic Characterization

Infrared spectroscopy (FTIR) was performed using a Bruker Vertex 70 FTIR-spectrophotometer (Bruker Optics Inc., Billerica, MA, USA) in the range of wavenumber between 4000–400 cm^−1^, with a resolution of 2 cm^−1^ (32 scans collected). The infrared spectra were recorded in absorbance. Infrared spectra of the neat uncured UPIR resins were carried out by spreading the liquid mixture on the KBr pellet, while for the cured UPIR resins, the spectra were collected by dispersing powder of the samples in KBr pellets.

### 2.5. Electrical and Electromagnetic Characterization

The electromagnetic characterization of the resins was performed on disk-shaped specimens (with a diameter of 5 cm and a thickness of 2 mm) using the impedance analyzer QuadTech (7600 Precision LCR Meter, Model B, QuadTech Inc., Marlborough, MA, USA). A suitable cell measurement [27] was used with the QuadTech impedance analyzer to obtain the impedance measurements. The measurements were carried out three times, fixing 25 points in frequency acquisition, each one obtained as the average value of 5 measurements at a fixed 5 V of voltage amplitude stimulus, with medium accuracy corresponding to an uncertainty of 0.5% on the measured data.

The data were obtained as module *Z(ω)* and phase *θ(ω)* of the impedance for each considered frequency, where *ω* = 2π*f* is the frequency *f* expressed in rad/sec. From these values, the complex impedance $\dot{Z}\left(\omega \right)$ is calculated as

(2) $$\dot{Z}\left(\omega \right)=Z\left(\omega \right)cos\left[\theta \left(\omega \right)\right]+i*Z\left(\omega \right)sin\left[\theta \left(\omega \right)\right]$$

where *i* is the imaginary unit.

By assuming a parallel RC model for the sample under test, the complex admittance $\dot{Y}\left(\omega \right)=1/\dot{Z}\left(\omega \right)$ could be expressed as

(3) $$\dot{Y}\left(\omega \right)=\frac{1}{R\left(\omega \right)}+i\omega C\left(\omega \right)$$

where *R* and *C* are the resistance and the capacity associated with a cylindrical geometry of section S and altitude d, corresponding to the electrode section and sample thickness, respectively. The real and imaginary part of the complex admittance, jointly with the geometry of the sample, and of the electrode configuration are therefore considered in order to derive the effective electrical conductivity *σ(ω)*, and the real $\varepsilon^{\prime }\left(\omega \right)\;$and imaginary $\varepsilon^{\prime \prime }\left(\omega \right)$ part of the complex permittivity, as follows:

(4) $$\sigma \left(\omega \right)=\frac{1}{R\left(\omega \right)}\times \frac{d}{S}=Re\left\{\dot{Y}\left(\omega \right)\right\}\times \frac{d}{S}$$

(5) $$\varepsilon^{\prime }\left(\omega \right)=Im\left\{\dot{Y}\left(\omega \right)\right\}\times \frac{d}{S\omega \varepsilon_{0}}$$

(6) $$\varepsilon^{\prime \prime }\left(\omega \right)=\frac{\sigma \left(\omega \right)}{\omega \varepsilon_{0}}$$

where *ε*_0_ = 8.854 × 10^−12^ F/m is the permittivity of the vacuum.

Finally, the ratio between the imaginary and real part of the complex dielectric permittivity leads to obtaining the loss tangent, *tanδ*, the characteristic element for an insulating material that must be as low as possible in frequency range as wide as possible:

(7) $$tan\delta \left(\omega \right)=\frac{\varepsilon^{\prime \prime }\left(\omega \right)}{\varepsilon^{\prime }\left(\omega \right)}$$

## 3. Results and Discussion

### 3.1. Thermal and Mechanical Characterization Results

The thermal characterization of the three commercial polyester imide resins was performed to compare the thermal properties of the analyzed electrical insulating materials and to select the resin showing the best thermal performance.

**Figure 2:**
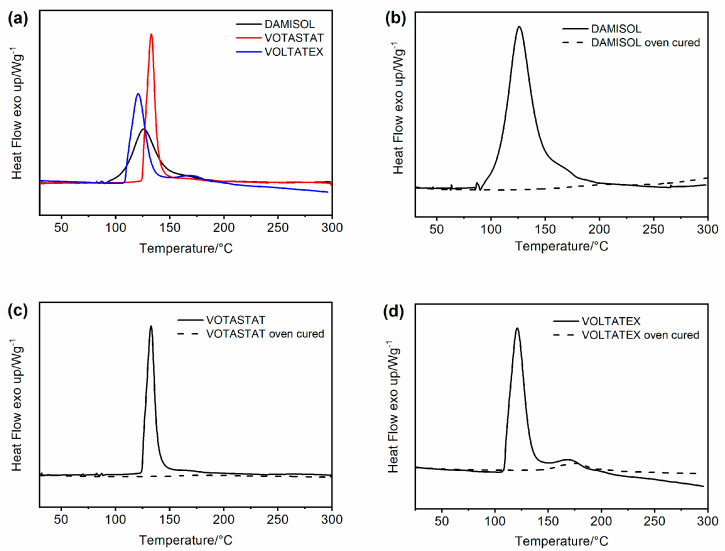
Comparison between the DSC curves of (**a**) uncured resins and (**b**–**d**) uncured (continuous curve) and cured (dashed curve) resins for each UPIR.

Figure 2 and Table 3 show the DSC curves and data, respectively, for each formulated sample. In particular, Figure 2a compares the curves obtained by performing the DSC measurements on the liquid uncured resins. For each sample, it is possible to detect the exothermic peaks due to the reaction heat developed during the polymerization process. The curves related to DAMISOL and VOTASTAT resins (see black and red curves in Figure 2a, respectively) present a single exothermic peak, while the VOLTATEX resin is characterized by the presence of two exothermic peaks (see blue curve in Figure 2a): the first in a temperature range between 105 and 150 °C, and the second one around 170 °C. These two peaks can represent two independent cure reactions [41]. In general, the curing of an unsaturated polyester resin (UPR) is a free radical polymerization in which the resin is transformed from the liquid state into a rigid crosslinked molecular structure, and to initiate the reaction, a source of free radicals is needed, using heat or a catalytic system [41]. Usually, UPR cured with a promoter exhibits two exothermic DSC peaks, whereas the resins without a promoter show only a single exothermic DSC peak [42,43]. However, a single exothermic peak has been observed for systems containing a nontoxic active crosslinking and effective initiator–inhibitor monomer, such the environmentally friendly polyester-imide resins developed by Xia et al. [33]. The comparison among DSC curves in Figure 2a allows observing the difference between the temperatures of starting polymerization (Tsp), corresponding to the left limit of the DSC exothermic peak. This temperature is an important parameter to evaluate for the industrial application, as it strongly influences the hardening conditions and, consequently, the costs of the impregnation process. For all the analyzed thermosets, the maximum curing temperature is between 120 and 135 °C; however, as reported in Table 3, DAMISOL resin shows the lowest value of Tsp, corresponding to about 86.1 °C. This last aspect allows stating that the DAMISOL resin achieves a curing degree of 100% with a curing process briefer than the other two thermosetting resins (see Table 2).

Figure 2b–d compares the DSC curves of the liquid uncured resin (continuous curve) and the oven-cured resin (dashed curve) for each sample. This comparison highlights that the hardening conditions proposed by the technical data sheets of the materials are suitable for making the resins reach a satisfactory degree of polymerization. In particular, concerning the resins DAMISOL and VOTASTAT (see Figure 2b,c, respectively), it can be observed that in the DSC profiles of the oven-hardened resins (see dashed curves), the exothermic peak disappears (see black and red dashed curves). These results evidence that a Cure Degree (DC) of 100% is obtained, as also shown in Table 3. VOLTATEX resin has a different DSC behavior, giving an oven-hardened material curve (see blue dashed curve in Figure 2d) that still shows an exothermic peak placed at a higher temperature, between 150 and 200 °C, indicating a partial curing degree (93.3%). The adopted hardening cycle conditions do not allow a complete polymerization of the resin. To obtain a curing degree of 100% for the VOLTATEX resin, it is necessary to increase the curing cycle condition (time and temperature). In any case, the treatment cycles used allow a degree of cure greater than 90%. The differences found in the DSC analysis, albeit small, are also found in the mechanical analyses, shown in Figure S2 of the Supplementary Materials. The found variations in the mechanical parameters are small. In particular, the Young modulus, the stress, and the strain at break range between 1 and 1.5 GPa, 15 and 25 MPa, and 2 and 4%, respectively. In particular, the resin with a higher curing degree presents a similar Young modulus (DAMISOL and VOTASTAT) but higher than that obtained for resin with a lower curing degree (VOLTATEX). The lower curing degree of the VOLTATEX resin creates a more flexible structure of the resin, causing an increase of strain at break.

**Figure 3:**
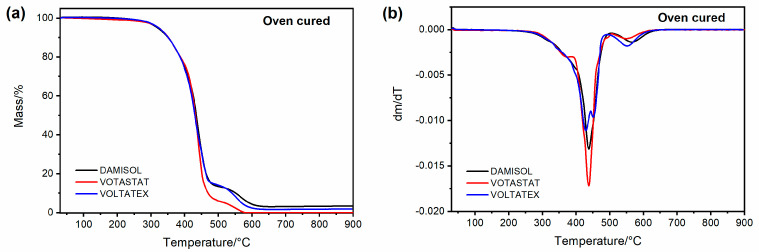
(**a**) TGA curves of oven-cured UPIR; (**b**) DTGA curves of oven-cured UPIR.

Figure 3a,b shows the TGA and the derivative mass loss curves (DTGA), respectively, as a function of temperature, for the polymerized samples. The measurements were performed in air flow, to evaluate the thermal performance of the samples in an environment similar to the operating conditions.

Thermal degradation behavior is essential to characterize a material’s thermal stability [33]. Table 4 shows the values of T_d5%_ and T_d50%,_ which indicate the temperature corresponding to a mass loss of 5 wt% and 50 wt%, respectively. T_d5%_ is defined as the initial degradation temperature, and it is usually considered to evaluate the thermal degradation stability of a material [18,33,44,45]. The TGA e DTGA profiles highlight that the three resins show thermal degradation behavior characterized by two stages, a first relevant step that realizes around 430 °C, and a second thermal degradation event, between 480 and 600 °C. All the samples show similar values of T_d5%_, about 320 °C, suggesting that all resins are characterized by good thermal stability. As reported in the literature for similar systems, this high thermal stability can be due to the imide molecular chain segments and the highly crosslinked molecular structure [33].

### 3.2. Spectroscopic Characterization

**Figure 4:**
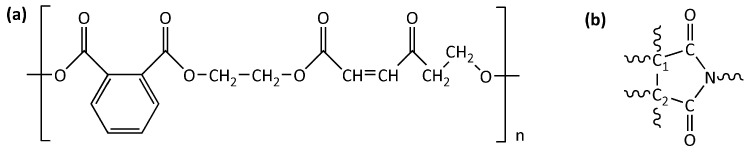
Chemical structure of (**a**) unsaturated polyester resin; (**b**) five-membered imide ring.

FT-IR investigation was performed to monitor the curing reaction of the unsaturated polyester imide resins. An unsaturated polyester resin has the general structure shown in Figure 4a [2,46], while an unsaturated polyester imide resin shows a similar structure but also contains five-membered imide rings in the chain, with the general formula shown in Figure 4b, in which C_1_ and C_2_ could be part of an aromatic system [47]. The C=C instaurations of the chain are involved in the reaction that generates the crosslinked network, and they are often employed to monitor the advancement of the thermosetting process of the analyzed resin composition [48].

The FT-IR spectra of the investigated resins display the absorbances characteristic of the described structures, as reported for the DAMISOL resin.

**Figure 5:**
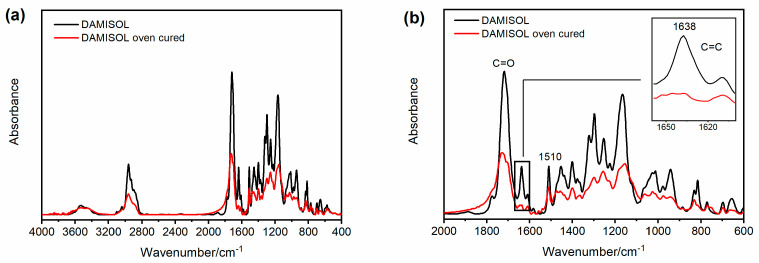
FT-IR spectra of DAMISOL resin: (**a**) range of wavenumber between 4000 and 400 cm^−1^; (**b**) range of wavenumber between 2000 and 600 cm^−1^.

**Figure 6:**
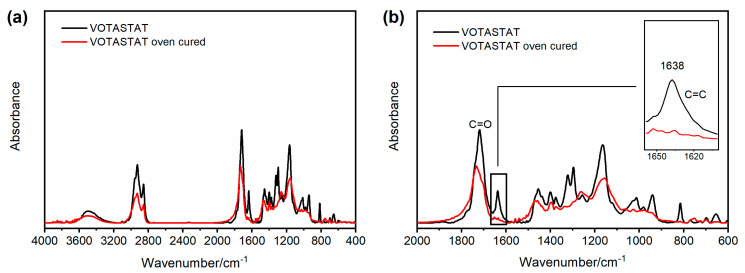
FT-IR spectra of VOTASTAT resin: (**a**) range of wavenumber between 4000 and 400 cm^−1^; (**b**) range of wavenumber between 2000 and 600 cm^−1^.

**Figure 7:**
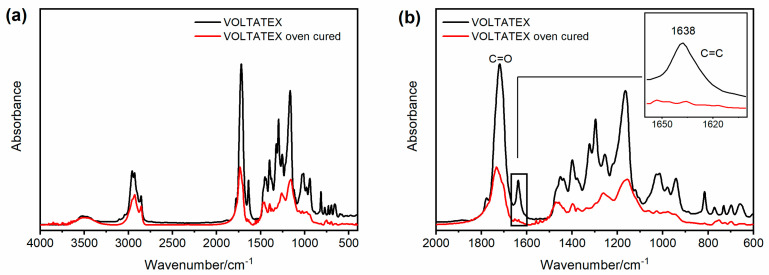
FT-IR spectra of VOLTATEX resin: (**a**) range of wavenumber between 4000 and 400 cm^−1^; (**b**) range of wavenumber between 2000 and 600 cm^−1^.

Figure 5 compares the FT-IR spectrum of the liquid uncured DAMISOL resin (see black curve) and the spectrum of the same oven-cured material (see red curve). Figure 5a displays the two spectra in the whole range of wavenumber, while Figure 5b focuses on the range between 2000 and 600 cm^−1^. Both the samples show strong absorption at 1510 cm^−1^, assigned to the vibrations of phenyl rings in the polymer backbone [46] and the signal of the carbonyl group stretching vibration. This last absorption includes the C=O bond of ester groups and imide functions. In particular, for the uncured sample (see black curve), the carbonyl band is a single broad signal around 1720 cm^−1^, while for the oven-cured sample (see red curve), the signal has a higher peak around 1725 cm^−1^ (imide C=O) with a shoulder at lower wavenumbers, at about 1703 cm^−1^, belonging to the ester C=O [49]. In the spectrum of the liquid sample also appears the band belonging to the C=C double bond stretching, at about 1638 cm^−1^, which is absent in the spectrum of thermally cured resin, as it is well detectable in the inset of Figure 5b. As already anticipated, the disappearance of this band provides experimental evidence that the resin composition is crosslinked to produce the thermoset polymer [48]. It is worth noting that similar spectroscopic results are also observable in the FT-IR spectra performed on the other two resin samples, as reported in Figure 6 and Figure 7.

### 3.3. Electrical and Electromagnetic Characterization

**Figure 8:**
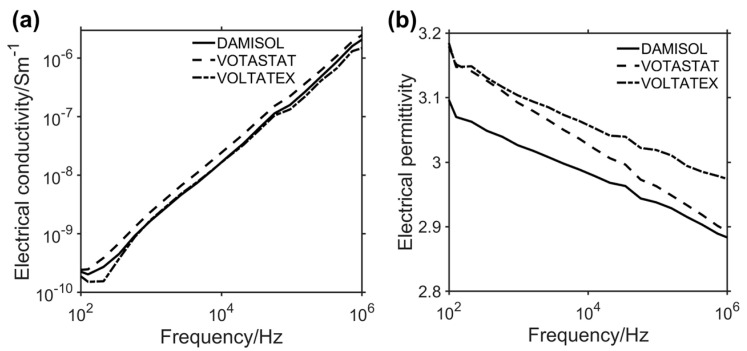
Broadband characterization of DAMISOL (continuous curves), VOTASTAT (dashed curves), and VOLTATEX (dash-dotted curves) resins in the range from 100 Hz to 1 MHz: (**a**) electrical conductivity; (**b**) electrical permittivity.

The broadband electromagnetic characterization of the three formulated materials in the frequency range from 100 Hz to 1 MHz allows for obtaining their complex electrical permittivity, from which the dependence of the electrical conductivity and the relative permittivity on the frequency can be derived. Figure 8a,b shows the dependence on the frequency of the electrical conductivity (Figure 8a) and the relative permittivity (Figure 8b).

**Figure 9:**
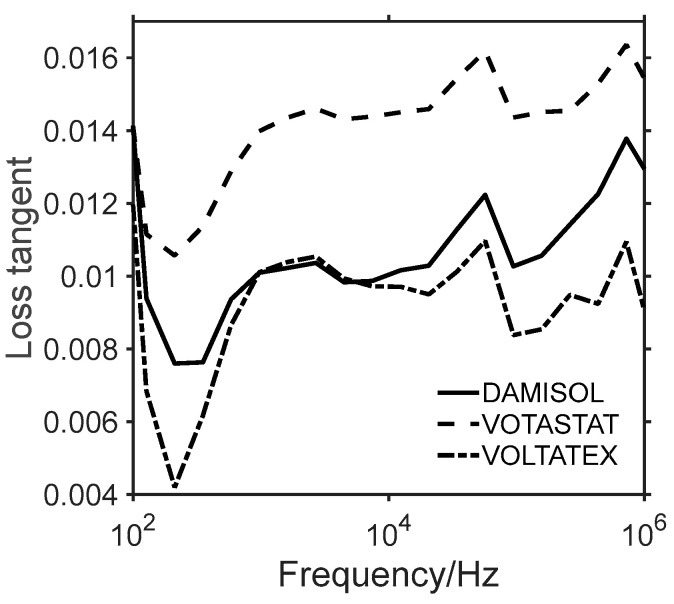
Loss tangent from 100 Hz to 1 MHz of DAMISOL (continuous curves), VOTASTAT (dashed curves), and VOLTATEX (dash-dotted curves) resins.

The real part of the relative permittivity is also referred to as the dielectric constant. It is a measure of the ability of a material to store electric energy by polarization. The imaginary part of the relative permittivity is also known as the dielectric loss factor and quantifies the losses associated with the polarization. From this component, the electrical conductivity is derived (Equation (4)). In the analyzed frequency ranges, dipolar polarization is observed, where a typical relaxation behavior occurs [50]. Data reported here evidence that all analyzed resins act as good insulators, with an electrical conductivity approaching a value of 10^−10^ S/m at 100 Hz. VOLTATEX resin is the more insulating one, with lower electrical conductivity. VOTASTAT exhibits higher electrical conductivity, which should be considered as the effect of a higher quantity of trapped charges in the amorphous phase [51], yet still remaining close to other resins and, therefore, in a range of values valid for insulation applications. In terms of electrical permittivity, data in Figure 8b show that, for all the analyzed resin formulations, a decreasing behavior with increasing frequency is observed, consistent with the behavior of similar systems [22,52]. DAMISOL resin performs better than the other resins, especially at the lower analyzed frequency value (Figure 8b), showing in this range the lowest electrical permittivity. For this material, the relative permittivity value at the lower analyzed frequency is 3.07 ± 0.01, whereas the VOTASTAT and the VOLTATEX resins assume the values of 3.15 ± 0.02 and 3.14 ± 0.02, respectively. Equation (7) allows obtaining the frequency behavior of the loss tangent (Figure 9) that helps to investigate the predominant conduction mechanisms in the observed frequency range.

The loss tangent, called the dissipation factor, maintains a value lower than 0.02 in the wide analyzed frequency range for all commercial resins. The maximum detected value is given for VOTASTAT resin at 723.2 kHz, where the loss tangent assumes the value of 0.016 ± 0.001. This is an excellent value for electrically insulating materials. It guarantees a very low energy dissipation, even up to several multiple switching frequencies, in applications such as secondary insulation systems in PWM feed motors.

**Figure 10:**
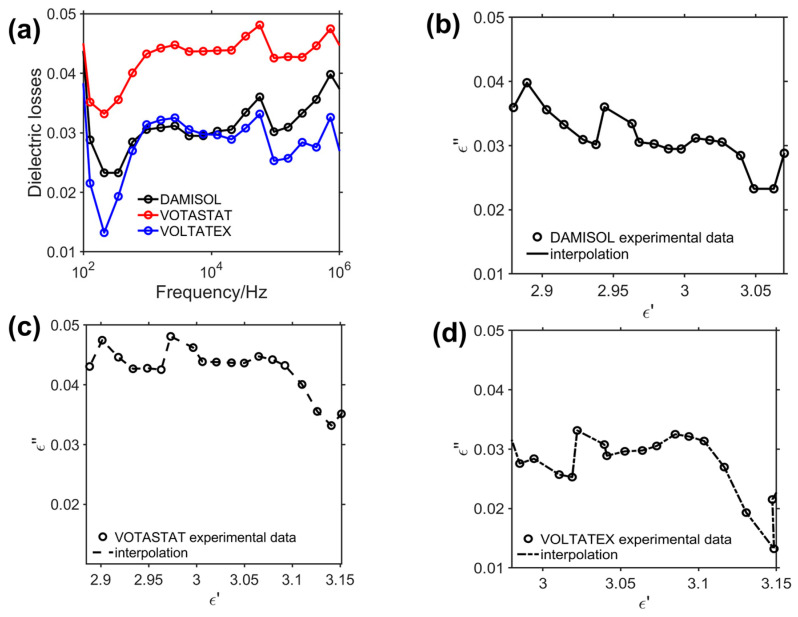
Dielectric losses of the considered resins (**a**) with corresponding Cole–Cole plots for real and imaginary parts of the complex permittivity: DAMISOL (**b**), VOTASTAT (**c**), and VOLTATEX (**d**).

The high increase of *tanδ* at lower analyzed frequencies suggests the presence of interfacial polarization mechanisms (Maxwell-Wagner) for all the considered resins, with a slower increasing behavior for the VOTASTAT case. Conversely, for a higher frequency, a dipolar orientation polarization starts to be predominant, as it is typical for this kind of material [49]. This behavior is confirmed by looking at the imaginary part of the complex permittivity (Equation (6)) representing the dielectric losses (Figure 10a) of the three considered commercial resins. If the Cole–Cole plot of the imaginary and real part of the complex permittivity of the three resin is considered, a non-circular behavior is detected. The resins manifest a non-Debye material behavior [53,54]. The absence of circular plots in the spectra is indicative of beta relaxation phenomena and the presence of interacting dipoles [50].

From the reported data on the losses, it is possible to observe that the VOLTATEX formulation can guarantee lower thermal dissipation between the compared insulation systems and is a good candidate for the insulation material in high PWM switching frequency. Furthermore, DAMISOL performs similarly to VOLTATEX from 1 kHz to 100 kHz, representing a good alternative for an insulation system in PWM-fed motors with limited switching frequency.

## 4. Conclusions

Electromagnetic characterization evidences an electrical behavior suitable for electrical insulation applications for all considered resins. In particular, VOLTATEX exhibits lower loss factor and electrical conductivity, whereas VOTASTAT shows the highest value for these parameters, and DAMISOL resin demonstrates an intermediate behavior. For this last material, a very good performance is manifested concerning its relative permittivity values. Furthermore, thermal characterizations highlight that, among the analyzed materials, the most promising for VPI industrial applications is DAMISOL resin, which shows good thermal stability, a high curing degree, and more cost-efficient processability. Therefore, this commercial system seems to fulfil thermal and energy-saving requirements, also being a good candidate as impregnating resin in secondary insulation material applications for inverted-fed motors.

## Figures and Tables

**Table 1:** Commercial information on the resins.

Resin	Trade Name	Company
**DAMISOL**	Damisol^®^ 3630 HTP 02 600	Von Roll SA, 69330 Meyzieu, France
**VOTASTAT**	VOTASTAT^®^ 5000	Gamma S.p.A., 29022 Bobbio (Piacenza), Italy
**VOLTATEX**	Voltatex^®^ 4200	Axalta Coating Systems, GmbH & Co. 42285 Wuppertal, Germany

**Table 2:** Curing cycle conditions of the commercial resins.

Resin	Polymerization Conditions (Time and Temperature)
**DAMISOL**	2 h at 150 °C
**VOTASTAT**	16 h at 140 °C
**VOLTATEX**	2 h at 130 °C + 1 h at 150 °C

**Table 3:** Results of DSC analysis.

Resin	T_sp_ (°C)	Δ*H_T_* (Jg^−1^)	Δ*H_Res_* (Jg^−1^)	Curing Degree DC (%)
**DAMISOL**	86.1	219.0	0.0	100
**VOTASTAT**	114.9	181.3	0.0	100
**VOLTATEX**	104.9	209.1	13.9	93.3

**Table 4:** Results of TGA analysis.

Resin	T_d5%_ (°C)	T_d50%_ (°C)	Residue at 900 °C (%)
**DAMISOL**	319.3	434.7	3.1
**VOTASTAT**	322.9	431.9	0.0
**VOLTATEX**	322.4	430.0	1.7

## Data Availability

Not applicable.

## Supplementary Materials

The following supporting information can be downloaded at: https://www.mdpi.com/article/10.3390/polym15061374/s1, Figure S1: Pictures of the obtained samples with the different commercial resins; Figure S2: Tensile test data relating epoxy resins DAMISOL, VOTASTAT and VOLTATEX: (a) stress–strain curves, (b) Young modulus values; (c) stress at break values; (d) strain at break values.
