# Supplementary material for: Thermal and Electrical Characterization of Polyester Resins Suitable for Electric Motor Insulation
Source: Polymers (Basel). 2023 Mar 9;15(6):1374. doi: 10.3390/polym15061374 (PMC10053653; doi:10.3390/polym15061374)
Supplement: Supplementary file 1 [file polymers-15-01374-s001.zip › polymers-2223492-supplementary.pdf]

Figure S1 shows the pictures of the analysed commercial resins after the curing stage.

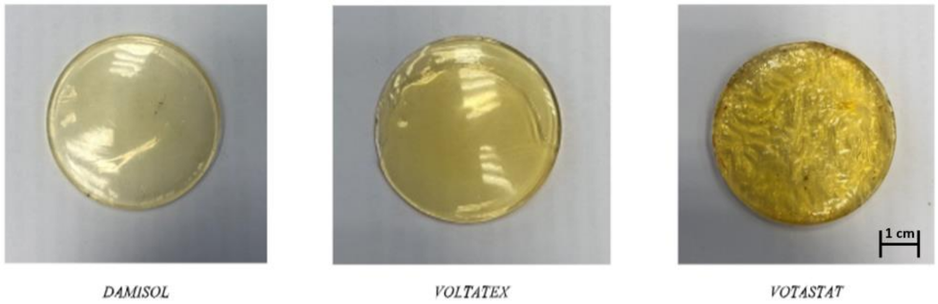

Figure S1. Pictures of the obtained samples with the different commercial resins.

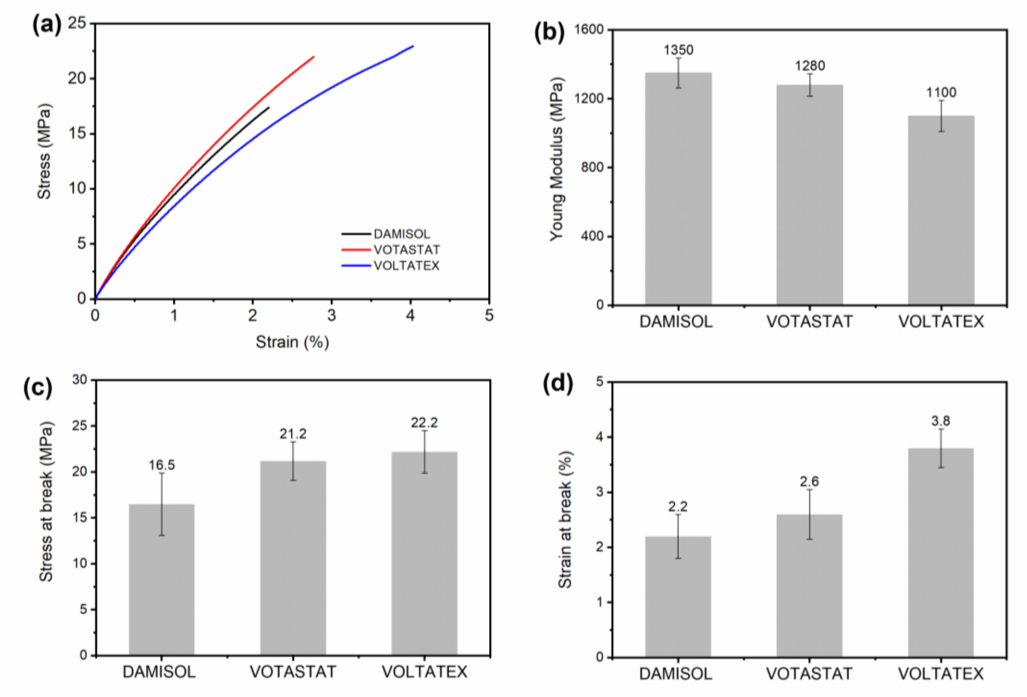

Figure S2. Tensile tests data relating epoxy resins DAMISOL, VOTASTAT and VOLTATEX: a) stress-strain curves, b) Young modulus values; c) stress at break values; d) strain at break values.
